# Exposure to domestic abuse and the subsequent risk of developing periodontal disease

**DOI:** 10.1016/j.heliyon.2022.e12631

**Published:** 2022-12-23

**Authors:** Sonica Minhas, Rachel Qian Hui Lim, Devan Raindi, Krishna M. Gokhale, Julie Taylor, Caroline Bradbury-Jones, Siddhartha Bandyopadhyay, Krishnarajah Nirantharakumar, Nicola J. Adderley, Joht Singh Chandan

**Affiliations:** a Institute of Applied Health Research, College of Medical and Dental Sciences, University of Birmingham, B152TT, United Kingdom; b Barts and the London School of Medicine and Dentistry, Queen Mary University of London, E1 2AD, United Kingdom; c Periodontal Research Group, School of Dentistry, Institute of Clinical Sciences, University of Birmingham, B152TT, United Kingdom; d School of Nursing, College of Medical and Dental Sciences, University of Birmingham, Birmingham Women's and Children's Hospitals NHS Foundation Trust, Birmingham, B152TT, United Kingdom; e The Department of Economics, University of Birmingham, B152TT, United Kingdom

**Keywords:** Domestic abuse, Periodontal disease, Periodontitis, Gingivitis

## Abstract

**Aims:**

Periodontal disease and domestic abuse (DA) are significant public health problems. Previous cross-sectional evidence indicates an association between DA exposure and development of periodontal disease. There have been no large-scale cohort studies exploring this relationship in a UK-setting. Our aim was to conduct a population-based retrospective open cohort study to explore the association between DA exposure and the subsequent development of general practitioner (GP)-coded periodontal disease.

**Materials and methods:**

We undertook a retrospective open-cohort study using the IQVIA Medical Research Database (IMRD) UK database between the 1^st^ January 1995 to 31^st^ January 2021. Women (aged 18 years and over) exposed to DA were matched by age, deprivation, and smoking status to up to 4 unexposed women, all of whom had no pre-existing record of periodontal disease. Cox regression analysis was used to calculate crude and adjusted hazard ratios (HRs) to describe the risk of developing periodontal disease in the exposed group.

**Results:**

23429 exposed patients were matched to 69815 unexposed patients. During the study period, 78 exposed patients had developed GP-recorded periodontal disease compared to 154 in the unexposed group, translating to an IR of 94.18 per 100,000 person years (py) and 54.67 per 100,000 py respectively. Following adjustment for key covariates, this translated to an aHR of 1.74 (95% CI 1.31–2.32), which was robust during our sensitivity analysis.

**Conclusions:**

Our results provide further evidence that DA exposure is associated with increased risk of developing periodontal disease. There is a need for swift implementation of public health policies to improve surveillance, reporting, and prevention of DA.

## 1 Introduction

Periodontal disease refers to a group of chronic inflammatory conditions of the gingiva, bone, and other supporting appendages of the teeth (Kinane et al., 2017), including gingivitis and periodontitis. Despite substantial progress in oral health globally, periodontal disease continues to be a significant public health challenge amongst adults. Latest estimates suggest that globally 10% of people have severe periodontitis (Bernabe et al., 2020) and in the UK, the last UK Dental Health Survey, reported that 45% of the population are affected by periodontitis (Steele & O’ Sullivan, 2009).

Gingivitis, localised inflammation of the gingiva, is the first manifestation of periodontal disease, and is induced by dental plaque accumulation (Kwon et al., 2021), also known as dental biofilm. Biofilm is composed of food residue, saliva, calcium salts and commensal bacteria. As biofilm maturation takes place, an inflammatory response is initiated by the host, presenting as gingival inflammation. In susceptible individuals, the inflammatory lesion, that in gingivitis is contained to the soft tissue may lead to progressive destruction of the gingiva, alveolar bone, periodontal ligaments (Kinane et al., 2017) and potentially abscess formation and tooth loss-this disease being called periodontitis. Furthermore, the literature reports positive associations between periodontal disease and systemic health conditions, with the evidence base continuing to expand (Bartold, 2012; Beck et al., 2019; Cullinan and Seymour, 2013; Scannapieco and Cantos, 2016). Periodontal disease has been linked to cardiovascular disease, including myocardial infarction, diabetes mellitus, rheumatoid arthritis, preterm birth and low-birth weight (Bartold, 2012; Cullinan and Seymour, 2013; Scannapieco and Cantos, 2016). Whilst causation has not been proved and the exact mechanisms underpinning these associations remaining unclear, but likely to involve extension of specific oral bacteria and/or inflammatory mediators into the systemic circulation, the high prevalence of periodontitis may translate into a significant general health risk and burden.

The aetiology of periodontal disease is multi-factorial, involving interactions between the dental microbiota, environment, and host inflammatory response (Dahlen et al., 2020; Kinane et al., 2017; Meyle and Chapple, 2015). Several risk factors for periodontitis and gingivitis have been identified, including genetic, cigarette smoking, systemic disease and socioeconomic factors, including education level (Bergstrom, 1989; Kinane et al., 2006; Kinane et al., 2017). Psychosocial factors have also been associated to varying degrees with periodontal disease (Croucher et al., 1997; Green et al., 1986; Segura Marcenes and Sheiham, 1992), although it is yet to be determined if stress exerts this effect through psychoneuroimmunological mechanisms and/or reduction in health behaviours/increases in destructive lifestyle (e.g., smoking, alcohol) (Robert J Genco et al., 1998).

Previous studies have suggested an association between exposure to abuse and poor oral health behaviours and outcomes (Kamimura et al., 2014; Kundu et al., 2014). However, these are limited by small sample size and cross-sectional design. To the best of our knowledge, there have been no large-scale cohort studies examining the association between domestic abuse (DA) and periodontal disease in an adult population. This is an important gap in the literature, given the prevalence of both DA and periodontal disease. Our aim was to conduct a retrospective open-cohort study to explore the relationship between DA and general practitioner (GP)-coded periodontal disease.

## 2 Materials and Methods

We conducted a population-based, retrospective, open, cohort study using the IQVIA Medical Research Database (IMRD) UK database to examine the risk of subsequent periodontal disease following exposure to domestic abuse in female patients between 1^st^ January 1995 to 31^st^ January 2021. IMRD-UK, previously called The Health improvement Network (THIN) database, consists of electronic medical records from over 800 general practices in the UK, who use the Vision software system. It is nationally representative of the UK's demographic structure and common comorbidities in the population (Blak et al., 2011).

Patient information is recorded in IMRD using a hierarchical coding system, called Read codes (Booth, 1994), which captures patient symptoms, examinations, and diagnoses. Other data available in IMRD include demographic information, prescriptions, and mortality data. To reduce the risk of under-reporting and ensure accurate coding, general practices were only included from the later of the two dates: one year following the date the practice began using the electronic medical records system, or the date the practice achieved acceptable mortality recording (Maguire et al., 2009).

The aim of this study was to explore the risk of developing GP-coded periodontal disease (periodontitis or gingivitis) in adult women (aged 18 years and above) with a recorded exposure to domestic abuse, compared to women with no recorded exposure. For this study, we have used Read codes to define the exposure (domestic abuse) and outcome of interest (periodontal disease; see AppendixA). The dataset and these code lists have been previously used to explore the life course of those experiencing domestic abuse and those experiencing oral disease (Chandan et al., 2021; Chandan et al., 2021; Chandan et al., 2020; Chandan et al., 2019; Chandan et al., 2020; Chandan et al., 2019; Zemedikun et al., 2021).

Exposed patients were matched to up to four unexposed patients, by age, Townsend deprivation quintile, and smoking status from the same General Practice. Each exposed patient was assigned an index date corresponding to the earliest date of recorded exposure (incident cases), or the date they became eligible to enter the study if the exposure preceded the study start date (prevalent cases). To reduce immortality time bias, the same index date was assigned to their matched unexposed counterparts (Lévesque et al., 2010). The exit date for all patients was defined as the earliest of the following dates: patient died, patient left the practice, last data collection from participating practice, study end date, or patient was diagnosed with the outcome of interest: periodontal disease (periodontitis or gingivitis).

Data at study entry on body mass index (BMI), smoking status, Townsend deprivation quintile, and ethnicity was captured from the dataset and described using proportions. Crude incidence rates per 100,000 person years were calculated for each cohort. As the data satisfied the proportional hazard assumption, Cox regression analysis was used to calculate the crude and adjusted hazard ratio (HR) of periodontal disease in exposed versus unexposed women. HRs are presented with 95% confidence intervals and statistical significance assumed at p < 0.05. Covariates included in the adjusted analysis were BMI, smoking status, deprivation and age at study entry. A sensitivity analysis of incident only patients (where exposure to DA occurred during the study period) matched to unexposed patients was also carried out to assess the robustness of the findings. A separate cohort was extracted to facilitate this sensitivity analysis as the exposure codes only included 14 × 8.00 and 14XG.00, both of which indicated an incident event of DA.

## 3 Results

During the study period, 23429 women were identified as having a primary care recorded exposure to DA who were matched to 69815 unexposed patients. Baseline characteristics for exposed and unexposed patients are described in Table 1. Due to matching, baseline characteristics between both groups were similar and there were no significant differences.

**Table 1:** Baseline characteristics in those exposed and unexposed to domestic abuse.

	Exposed Group	Unexposed Group
**Number of Women (n)**	23429	69815
**Median (IQR) follow-up period (person years)**	2.47 (0.97–5.22)	3.12 (1.31–5.97)
**Mean (SD) age at cohort entry (years)**	37.16 (12.63)	36.78 (12.20)
**Body mass index, n (%)**		
Underweight (<18·5 kg/m^2^)	1044 (4.46)	2455 (3.52)
Normal (18·5–24·9 kg/m^2^)	8712 (37.18)	26994 (38.67)
Overweight (25·0–29·9 kg/m^2^)	5011 (21.39)	16060 (23.00)
Obese (>30·0 kg/m^2^)	4619 (19.71)	15213 (21.79)
Not available	4043 (17.26)	9093 (13.02)
**Smoking status, n (%)**		
Current smoker	10073 (42.99)	28575 (40.93)
Non-current smoker	12678 (54.11)	40346 (57.79)
Not available	678 (2.89)	894 (1.28)
**Townsend index, n (%)**		
(Least deprived) 1	2138 (9.13)	6643 (9.42)
2	2579 (11.01)	7397 (10.60)
3	3901 (16.65)	11435 (16.38)
4	5169 (22.06)	15244 (21.83)
5	52222 (22.29)	15151 (21.70)
Not available	4420 (18.87)	13945 (19.97)
**Ethnicity, n (%)**		
White	13066 (55.77)	35669 (51.09)
Black	894 (3.82)	2213 (3.17)
South Asian	1324 (5.65)	2909 (4.17)
Mixed	337 (1.44)	764 (1.09)
Other	580 (2.48)	1782 (2.55)
Missing	7228 (30.85)	26478 (37.93)

During the study, there were 78 incident outcomes of GP-recoded periodontal disease in the exposed group compared to 154 incident outcomes in the unexposed group, translating to an incidence rate (IR) of 94.18 per 100,000 person years (py) and 54.67 per 100,000 py respectively. Accounting for potential confounders (age, BMI, smoking status, and Townsend deprivation quintile at study entry), this corresponded with an adjusted hazard ratio (aHR) of 1.74 (95% CI 1.31–2.32; p < 0.001). Further details can be seen in Table 2.

**Table 2:** The risk of developing periodontal disease in those exposed and unexposed to domestic abuse.

	Periodontal Disease	
	Exposed	Unexposed
**Number of Incident Outcomes**	78	154
**Person-Years**	82820	281696
**Incidence Rate (per 100,000 person years)**	94.18	54.67
**Hazard Ratio (95% confidence interval)**†	1.71 (1.30–2.24)	
**p-value**	<0.001	
**Adjusted Hazard Ratio (95% confidence interval)**‡	1.74 (1.31–2.32)	
**p-value**	<0.001	

† Unadjusted hazard ratio.

‡ Adjusted hazard ratio: adjusted for age, Townsend deprivation quintile, BMI, and smoking status at study entry.

In the sensitivity analysis of incident only patients, 5461 exposed women were included and matched to 16369 unexposed women. Baseline characteristics, described in Table 3, were similar to the main cohort. Baseline characteristics were similar between the two groups in this cohort too, due to matching. In this cohort, 22 exposed patients had a GP-coded periodontal disease diagnosis (IR 115.64 per 100,000 py) compared to 35 controls (IR 56.18 per 100,000 py). This translated to an aHR of 2.17 (95% CI 1.25–3.77; p < 0.001). Further details can be seen in Table 4.

**Table 3:** Incident Only Cases; Baseline characteristics in those exposed and unexposed to domestic abuse.

	Exposed Group	Unexposed Group
**Number of Women (n)**	5461	16369
**Median (IQR) follow-up period (person years)**	2.57 (1.01–5.22)	3.05 (1.27–5.65)
**Mean (SD) age at cohort entry (years)**	37.73 (12.74)	37.28 (12.34)
**Body mass index, n (%)**		
Underweight (<18.5 kg/m^2^)	221 (4.05)	539 (3.29)
Normal (18.5–24.9 kg/m^2^)	2196 (40.21)	6505 (39.74)
Overweight (25.0–29.9 kg/m^2^)	1290 (23.62)	3815 (23.31)
Obese (>30.0 kg/m^2^)	1086 (19.89)	3644 (22.26)
Not available	668 (12.23)	1866 (11.40)
**Smoking status, n (%)**		
Current smoker	2209 (40.45)	6168 (37.68)
Non-current smoker	3197 (58.54)	10125 (61.85)
Not available	55 (1.01)	76 (0.46)
**Townsend index, n (%)**		
(Least deprived) 1	602 (11.02)	1973 (12.05)
2	670 (12.27)	1885 (11.52)
3	955 (17.49)	2752 (16.81)
4	1158 (21.20)	3467 (21.18)
5	1173 (21.48)	3411 (20.84)
Not available	903 (16.54)	2881 (17.60)
**Ethnicity, n (%)**		
White	2790 (51.09)	8372 (51.15)
Black	171 (3.13)	573 (3.50)
South Asian	342 (6.26)	822 (5.02)
Mixed	64 (1.17)	235 (1.44)
Other	121 (2.22)	454 (2.77)
Missing	1973 (36.13)	5913 (36.12)

**Table 4:** Incident Only Cases; The risk of developing periodontal disease during the study period in those exposed and unexposed to domestic violence and abuse.

	Periodontal Disease	
	Exposed	Unexposed
**Number of Incident Outcomes**	22	35
**Person-Years**	19024	62301
**Incidence Rate (per 100,000 person years)**	115.64	56.18
**Hazard Ratio (95% confidence interval)**†	2.04 (1.20–3.48)	
**p-value**	0.009	
**Adjusted Hazard Ratio (95% confidence interval)**‡	2.17 (1.25–3.77)	
**p-value**	0.006	

† Unadjusted hazard ratio.

‡ Adjusted hazard ratio: adjusted for age, Townsend deprivation quintile, BMI, and smoking status at study entry.

## 4 Discussion

This study provides further evidence describing an association between exposure to domestic abuse and the subsequent development of periodontal disease. Due to lack of cohort studies exploring this relationship, it is not easily possible to compare our findings with the existing literature.

However, there is evidence that demonstrates the ill-effect of domestic abuse (DA) on oral health status in general. A cross-sectional study by Kundu et al. explored oral health behaviours as well as oral health status in females in India affected by domestic violence compared with those not affected (Kundu et al., 2014). Their findings revealed a significantly higher proportion of non-DA patients reporting better oral hygiene behaviours (namely frequency of brushing). Furthermore, the clinical periodontal parameters investigated (loss of attachment and Community Periodontal Index) demonstrated worse periodontal status in the DA group.

The psychosocial impact of DA is likely to explain the findings of both previous studies and our own findings. Survivors of DA are likely to have undergone extreme stress which may account for reduced/ineffective oral hygiene practice as well as the possibility for an increase in negative lifestyle behaviours such as smoking and irregular dental visits. There is also evidence that coping strategies may be a significant determinant in the risk for periodontitis in individuals exposed to stress and therefore incorporating this into holistic management of DA patients is crucial, particularly as they may be likely to have been isolated from a support network (R.J. Genco et al., 1999).

The impact of providing oral healthcare in general to DA survivors has also been investigated in a longitudinal study carried out in the United States where dental residents were provided with tailored education to enable care at DA shelters. Dental care included periodontal therapy where required and the impact of the oral health program was measured by the Oral Health Related Quality of Life (OHRQoL) Questionnaire (Abel et al., 2013). By the end of the study participants demonstrated improved OHRQoL as well as high levels of satisfaction with the oral health program suggesting that dental treatment (including periodontal care) by trained dentists can have positive benefits within this population.

As one in three women are estimated to be affected by DA at least once in their lifetime (World Health Organization, 2021), our findings suggest that there may be a significant proportion of periodontal disease occurring in women with exposure to DA and oral health professionals are likely to encounter women with exposure to DA. This highlights a reminder for dentists to be aware of DA in their clinical practice among patients presenting with periodontal disease. Current evidence suggests screening is a poorly implemented practice among oral health professionals (Harris et al., 2016; Nelms et al., 2009), although the literature indicates that DA survivors would like their health care providers to ask about abuse (Fogarty et al., 2002; Macdonald, 2021; Nelms et al., 2009). Whilst there is insufficient evidence to support use of any particular screening tool, ample guidance has been provided by authorities for health care providers in the UK, including specifically for dental professionals (Department of Health and Social Care, 2017; NHS England and NHS Improvement, 2021). Inclusion of teaching on DA to the dental curriculum and within ongoing continuing professional development (CPD) is vital for improving the knowledge and competency of dentists to recognise DA and provide trauma-informed care to survivors, as well as supporting their access to healthcare and support services. Surveillance and appropriate screening are important facets of the public health response to DA, as they can improve the patient care of survivors and prevent further injuries or ill-health outcomes (World Health Organization, 2013). Furthermore, our study also provides additional evidence supporting the need for public health interventions to prevent DA and its negative downstream consequences on health.

Despite the findings, there are some important limitations to consider. It is well established that exposure to DA appears to be universally under-reported and therefore under-recorded (Chandan et al., 2020; Gracia, 2004). This is likely to result in a misclassification bias in terms of exposure assignment. Equally, despite its prevalence, there are yet to be any studies validating the nature of periodontal coding in primary care records also leading to a misclassification bias. However, there is nothing to suggest that the misclassification would be differential between the groups. Since the diagnosis of periodontal disease is primarily undertaken by dentists, the Read codes for periodontal disease are likely recorded following receipt of clinical letters from patients’ dental practitioner or self-reporting by the patient to the GP. However, GPs may also be able to diagnose periodontal disease since oral health is included in the undergraduate medical curriculum (Adam et al., 2021; Royal College of General Practitioners, 2022). In light of this, there is an urgent need to improve dental coding according to the 2017 periodontal disease classification system in primary care settings to support patient care and epidemiological research on periodontal disease (Adam et al., 2021). Future research should also utilise more recognised parameters of periodontal status including probing pocket depth, clinical attachment level, plaque scores and bleeding scores. The use of specific oral health related patient reported outcomes are also fundamental within such cohorts. Additionally, future work should explore the nature and quality of dental care provided to survivors in addition to clinical and patient reported outcomes. Another limitation is that DA has a broad definition, encompassing many forms of violence (Domestic Abuse Act 2021, 2021). Due to limitations of clinical coding, we were unable to explore the association between different subtypes of DA and the development of periodontal disease. There is scope for future research exploring this to determine if there is a dose-response relationship and to define the relationships between subtypes of abuse and periodontal disease.

In conclusion, our results provide new evidence that exposure to DA is associated with an increased risk of developing periodontal disease (periodontitis and/or gingivitis), coded by GPs. Our study highlights the need for further research on the association between DA exposure and periodontal disease, given the prevalence of both in the population and the systemic ill health burden associated with periodontal disease. We would also urge for an improvement in the clinical coding of dental conditions in primary care records. Urgent implementation of public health measures to prevent DA and its downstream consequences are essential.

## Clinical Relevance

### Scientific rationale for study

Previous cross-sectional evidence has shown an association between domestic abuse (DA) and periodontal disease. This association has not previously been tested in population-based cohort studies in a UK setting.

### Principal findings

Our results provide new cohort-based evidence in a UK setting of a relationship between exposure to domestic abuse and increased risk of subsequent GP-coded periodontal disease.

### Practical implications

Given the high prevalence of domestic abuse and periodontal disease, our results highlight the important role for oral health professionals in screening for domestic abuse and supporting survivors’ access to healthcare services.

## Ethical approval

Anonymised Data was used from the Data provider to the University of Birmingham. Use of IMRD is approved by the UK Research Ethics Committee (reference number: 18/LO/0441); in accordance with this approval, the study protocol must be reviewed and approved by an independent Scientific Review Committee (SRC). IMRD incorporates Data from The Health Improvement Network (THIN), A Cegedim Database. Reference made to THIN is intended to be descriptive of the Data asset licensed by IQVIA. This work will use de-identified Data provided by patients as a part of their routine primary care. For this project, an amendment to the approved SRC protocol 18THIN034 has been approved.

## Declarations

### Author contribution statement

Sonica Minhas, Rachel Qian Hui Lim, Devan Raindi, Julie Taylor, Caroline Bradbury-Jone, Siddhartha Bandyopadhyay, Krishnarajah Nirantharakumar, Nicola J Adderley: Analyzed and interpreted the data; Wrote the paper.

Joht Singh Chandan: Conceived and designed the experiments; Analyzed and interpreted the data; Wrote the paper.

Krishna M Gokhale: Contributed reagents, materials, analysis tools or data; Wrote the paper.

### Funding statement

This research did not receive any specific grant from funding agencies in the public, commercial, or not-for-profit sectors.

### Data availability statement

Data will be made available on request.

### Declaration of interest's statement

The authors declare no competing interests.

### Additional information

No additional information is available for this paper.

## Appendix A Supplementary data

Supplementary Material v1 Supplementary Material v1
