# Supplementary material for: Exposure to domestic abuse and the subsequent risk of developing periodontal disease
Source: Heliyon. 2022 Dec 23;8(12):e12631. doi: 10.1016/j.heliyon.2022.e12631 (PMC9813698; doi:10.1016/j.heliyon.2022.e12631)
Supplement: Supplementary Material v1 [file mmc1.docx]

Supplementary Material

Table of Contents

[Appendix A: Read codes for exposure and outcomes 2](#_Toc105273500)

# Appendix A: Read codes for exposure and outcomes

**Domestic Violence and Abuse**

| Clinical Code | Description |
| --- | --- |
| 14X3.00 | History of Domestic Violence |
| 14X8.00 | Victim of domestic violence |
| 14XD.00 | History of domestic abuse |
| 14XD000 | History of domestic emotional abuse |
| 14XD100 | History of domestic physical abuse |
| 14XD200 | History of domestic sexual abuse |
| 14XE.00 | History of being victim of domestic violence |
| 14XG.00 | Victim of domestic abuse |

**Periodontal Disease**

| Clinical Code | Description |
| --- | --- |
| 1928.00 | Bleeding Gums |
| 2552.00 | O/E- gingivitis |
| 2556.00 | O/E- bleeding gums |
| J03..11 | Gingivitis/gingival disease |
| J030.00 | Acute gingivitis |
| J031.00 | Chronic gingivitis |
| J031.11 | Gingivitis |
| J031000 | Simple marginal gingivitis |
| J031100 | Ulcerative gingivitis |
| J031200 | Desquamative gingivitis |
| J031300 | Hyperplastic gingivitis |
| J031z00 | Chronic gingivitis NOS |
| J03z.00 | Gingival and periodontal disease NOS |
| 2553.00 | O/E- pyorrhoea |
| J03..12 | Periodontal disease |
| J033.00 | Acute periodontitis |
| J033200 | Paradontal abscess |
| J033300 | Periodontal abscess |
| J033z00 | Acute periodontitis NOS |
| J034.00 | Chronic periodontitis |
| J034200 | Chronic periodontitis simplex |
| J034400 | Alveolar pyorrhoea |
| J034z00 | Chronic periodontitis NOS |
| J035.00 | Periodontosis |
| J051200 | Loss of teeth due to local periodontal disease |
